# Towards "biophysical psychiatry": a modeling approach for studying effects of schizophrenia-linked genes on single-neuron excitability

**DOI:** 10.1186/1471-2202-16-S1-P45

**Published:** 2015-12-18

**Authors:** Tuomo Mäki-Marttunen, Geir Halnes, Anna Devor, Aree Witoelar, Francesco Bettella, Srdjan Djurovic, Yunpeng Wang, Gaute T Einevoll, Ole A Andreassen, Anders M Dale

**Affiliations:** 1 NORMENT, Institute of Clinical Medicine, University of Oslo, Oslo, Norway; 2 Department of Mathematical Sciences and Technology, Norwegian University of Life Sciences, Ås, Norway; 3 Department of Neurosciences, University of California San Diego, La Jolla, San Diego, CA, USA; 4 Department of Radiology, University of California San Diego, La Jolla, San Diego, CA, USA; 5 Department of Medical Genetics, Oslo University Hospital, Oslo, Norway

## 

Genome-wide association studies (GWAS) employing large sample sizes and sophisticated statistical methods have recently yielded detailed information on the set of genes affected in various psychiatric disorders. This is especially important for polygenic, highly heritable disorders such as schizophrenia (SCZ) [1]. The success lately witnessed in gene discovery brings up the next big challenge for psychiatric genetics - translation of the genetic associations into biological insights [2]. In this study, we propose a computational approach for investigating the effects of a collection of excitability-related genes on various neuronal characteristics. As a proof of principle, we apply our approach for studying effects of SCZ-linked genes on firing behavior of a layer V pyramidal cell (L5PC).

A total of 108 genetic loci were recently confirmed to be associated with the risk of SCZ [3]. These loci span a wide set of protein-coding genes. The disorder is associated with genes affecting transmembrane currents of all major cationic species, Na^+^, K^+^, and Ca^2+^. In addition, some of the SCZ-linked genes are involved in regulation of the Ca^2+ ^concentration in the intracellular medium, which is another great contributor to neuron excitability. All above aspects of electrophysiology are included in a recent multi-compartmental model of L5PC [4], which accurately describes the perisomatic firing behavior and its interplay with the generation of an apical Ca^2+ ^spike. In this work, we rely on data from functional genomics studies that describe the effects of variants of certain ion channel or calcium transporter-encoding genes on the channel activation/inactivation properties or intracellular Ca^2+ ^dynamics. We carry out our study by linking these effects to a change in the corresponding neuron model parameters, and observing the implication that these variants have on the information integration in an L5PC.

It should be noted that information does not generally exist for the effect of single nucleotide polymorphism (SNP) variants identified through GWASs on the biophysical parameters required for the computational models. We instead use information obtained from *in vitro *studies of more extreme genetic variations, including loss of function mutations. A central assumption of this approach is that the effects of SNP variants can be represented as scaled-down versions of those of the more extreme variants, and that the emergence of the full psychiatric disease phenotype results from the combined effect of a large number of subtle SNP effects.

Our results show a multitude of alterations of the firing behavior and integration of apical stimuli in neurons equipped with the considered gene variants. An especially interesting observation is that the variants affect the suppression of a second apical stimulus, which might have a connection with the deficit in prepulse inhibition, a condition often observed in SCZ patients. Although the analyses presented here are specific to L5PCs and SCZ-related genes, our "biophysical psychiatry" framework may be directly applicable to other cell types and other polygenic diseases, such as bipolar disorder and autism, given an identification of risk genes related to neuronal excitability. Furthermore, our approach could be directly applied to biophysically detailed models of neuronal networks and extended to consider synaptic ion channel-encoding genes that are relevant in SCZ [5].
